# Surgery of Benign Ovarian Masses by a Gynecological Cancer Surgeon: A Cohort Study in a Tertiary Cancer Centre

**DOI:** 10.7759/cureus.9201

**Published:** 2020-07-15

**Authors:** Michela Quaranta, Rahul Nath, Gautam Mehra, Yasser Diab, Ahmad Sayasneh

**Affiliations:** 1 Gynaecological Oncology, Guy's and St Thomas' NHS Foundation Trust, London, GBR; 2 Gynaecology, Guy's and St Thomas' NHS Foundation Trust, London, GBR; 3 School of Life Course Sciences, Faculty of Life Sciences and Medicine, King's College, London, GBR

**Keywords:** multidisciplinary decision-making, diagnostic performance, false positive, ovarian neoplasm, cancer center

## Abstract

Objectives

This study aimed to evaluate diagnostic performance in characterising ovarian masses by our gynaecological oncology multidisciplinary team meeting (MDM). Surgical outcome and overall impact on patients and healthcare service were also assessed.

Methods

This was a prospective cohort study of all women with adnexal masses presenting to the gynaecological oncology MDM at a central London tertiary cancer centre between February 2017 and February 2018. The multidisciplinary team (MDT) outcome, imaging details, subjective opinion, tumour markers, surgical details, and final histological diagnosis were collected. Diagnostic performance was also determined.

Results

There were 200 eligible patients in the study period. MDM imaging review demonstrated a sensitivity of 98.4% (95% CI: 94.3% to 99.8%) and a specificity of 52% (95% CI: 40.2% to 63.7%). Thirty-five cases were false positive, either presumed invasive cancers (51%) or borderline tumours (49%). The most common histological types were serous (37%) and mucinous (31%) cystadenomas. A retrospective application of the International Ovarian Tumor Analysis (IOTA) Assessment of Different NEoplasias in the adneXa (ADNEX) model suggests a potential reduction in false-positive rates (17%). Among the false-positive cases, there was no postoperative (90 days) mortality and postoperative morbidity was 14% with only grade 2 (CD2) complications according to Clavien and Dindo's CD classification.

Conclusion

An MDT has high sensitivity but low specificity when characterising ovarian masses referred with possible ovarian cancer to the tertiary centre. False-positive values in ovarian cancers are an important indicator of over-treatment. More research is required to assess other methods, such as the IOTA ADNEX model, to reduce the false-positive value.

## Introduction

Ovarian malignancies are associated with significant morbidity and carry the worst prognosis and highest mortality of all the gynaecological cancers. Early identification is essential to improve survival rates and prognosis [1]. Diagnosis remains challenging due to the asymptomatic nature of early disease, late presentation in women, and limitations in imaging modalities for differentiating ovarian benign and malignant lesions [2].

The availability of pelvic imaging has significantly increased the detection of adnexal masses in both pre- and post-menopausal women. This was reported in the UK Collaborative Trial of Cancer Screening (UKCTOCS), estimating a 9% chance of an incidental finding of an ovarian mass in asymptomatic postmenopausal women [3]. The risk of malignancy in such cases is low, with an absolute risk of ovarian epithelial cancer of 1.08%; however, the risk of malignancy in symptomatic patients is expected to be higher. The National Institute of Health (NIH) estimates a woman’s lifetime risk of surgery for a suspected ovarian neoplasm is 5%-10% [4]. Pelvic ultrasound is increasingly used for a variety of clinical indications and its widespread use may constitute inadvertent screening.

In recent years, there has been a concerted effort to develop strategies to distinguish benign ovarian masses from those with invasive potential. Imaging algorithms have been developed and validated such as the Risk of Malignancy Index, the International Ovarian Tumor Analysis Group (IOTA) Simple Ultrasound Rules and the IOTA Assessment of Different NEoplasias in the AdneXa (ADNEX) model [2,5-6]. The IOTA ADNEX model is a risk prediction model that aims to reliably distinguish between benign, borderline, stage I invasive, stage II-IV invasive, and secondary metastatic adnexal tumours [6].

Whilst the treatment of ovarian cancer in specialised surgical centres is known to improve survival, we have become increasingly aware of the implications of the overtreatment of benign ovarian masses, including surgical complications, early menopause, and healthcare costs [7-8].

This study evaluated the gynaecological cancer centre’s multidisciplinary team meeting (MDM) diagnostic performance in characterising ovarian masses. This was undertaken by an analysis of the imaging features and histological subtypes of the benign adnexal masses that were falsely assumed malignant and consequently underwent surgical treatment. We also assessed the surgical outcomes of these false-positive cases.

## Materials and methods

This was a prospective, single-centre cohort study conducted at a central London tertiary cancer centre (Guy’s and St Thomas’ NHS Foundation Trust (GSTT)) between February 2017 and February 2018. Eligible women were those presenting with adnexal masses who were discussed at our gynaecological oncology MDM and who underwent surgical treatment under the oncology team. All cases were recruited consecutively from MDM. The study was approved as a service evaluation by the Trust's clinical governance committee.

Data included the following: MDM outcome, ultrasonography/magnetic resonance imaging (MRI)/computed tomography (CT) findings, radiology consultant's subjective impression, tumour markers, surgical details, operating times, intraoperative complications, postoperative morbidity using Clavien and Dindo's classification and 90 days mortality [9]. The NHS Caldicott Principles were adhered to in all steps of data collection and handling [10].

Only women with adnexal pathology confirmed on histology or those with follow-up imaging findings at a one-year interval were included in our study. Patients excluded were women in whom imaging did not detect an ovarian mass, women with a personal history of cancer, and those with a presumed diagnosis of benign lesions but with less than one year of follow-up.

The MRI variables collected included: restricted diffusion (Yes/No), soft tissue enhancement (Yes/No), multi-septation (Yes/No), suspicious lymph nodes (Yes/No), ascites (Yes/No), subjective opinion (benign, borderline, malignant), including, when given, a histological prediction (serous, mucinous, etc.). The final diagnosis was based on pathology and confirmed by a central histology review at MDM. Tumours were classified according to the World Health Organization (WHO) 2014 classification [11].

In order to calculate the diagnostic performance of the cancer centre's MDM, the outcomes of adnexal masses that were referred back to the referring units to be treated locally were retrieved. All histology was reviewed centrally to confirm the final diagnosis. For statistical analysis and to fit within the 2x2 contingency tables, borderline ovarian tumours were considered malignant. In cases managed conservatively, follow-up imaging was reviewed when available.

Although not within the primary remit of the study, for the purpose of secondary analysis, available ultrasound images and reports were reviewed on PACS Sectra (SECTRA IDS7 Version 20.2.6.3318, Sectra AB, Sweden) and Astraia (Astraia Version 1.24.10, Astraia Software GMBH, Germany) reporting systems by experienced ultrasound examiner (AS). The IOTA ADNEX model was calculated when the required parameters were retrievable retrospectively [2].

Statistical analysis

Diagnostic performance measures including sensitivity, specificity, and positive and negative likelihood ratios were calculated, 2 × 2 contingency values were obtained. Binomial exact 95% CI values were calculated for sensitivities, specificities, and positive and negative likelihood ratios. MedCalc v12.3.0 software (MedCalc Software, Mariakerke, Belgium) was used to calculate 95% CI when required.

## Results

**Figure 1:**
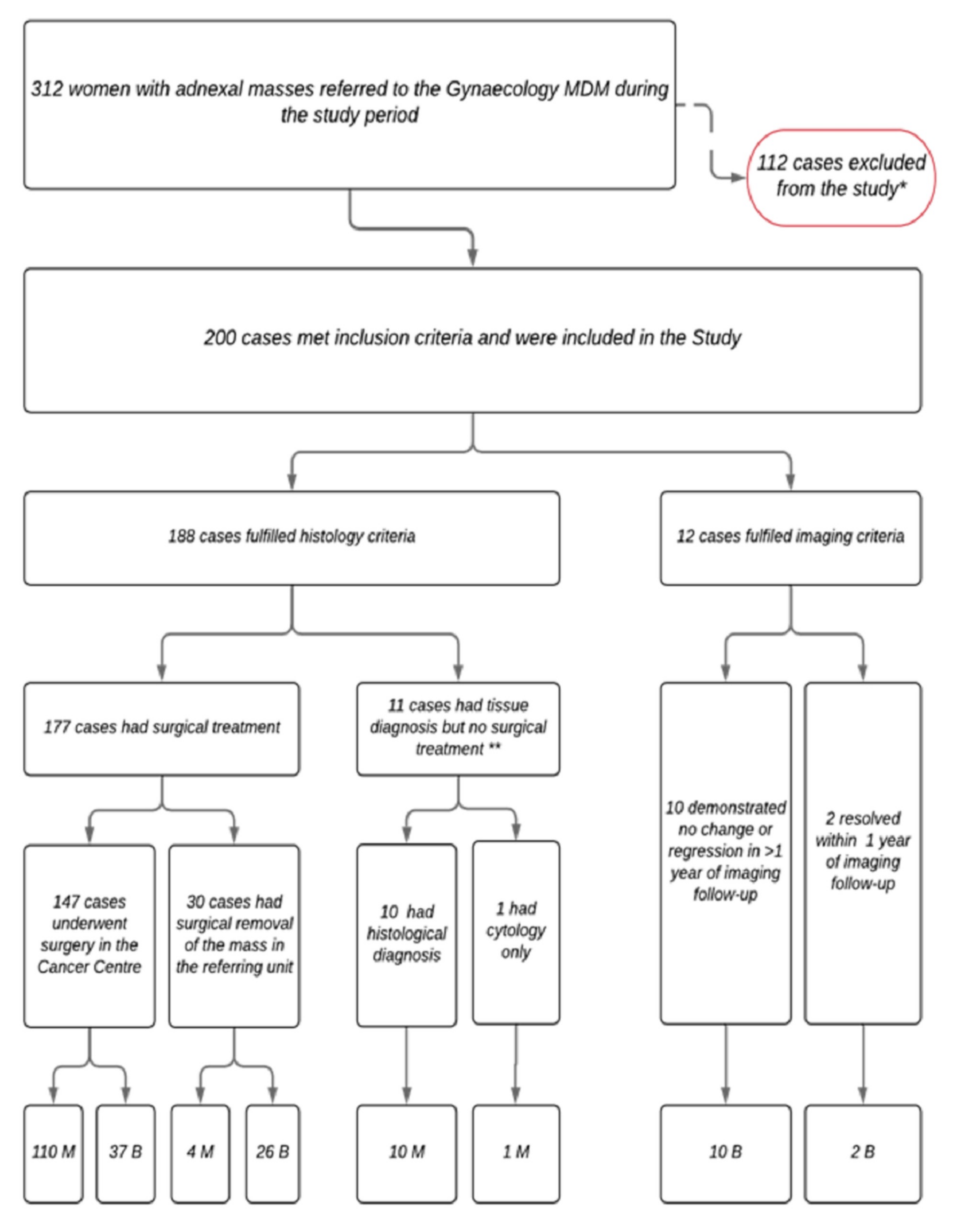
Distribution of women referred to the MDM during the study period * see text for cause of exclusion ** 8 had palliative chemotherapy, 2 were not fit for treatment, 1 case had cytology and received palliative chemotherapy MDM: multidisciplinary meeting; M: malignant; B: benign

A total of 312 women with adnexal masses were referred to the MDM during the study period. One-hundred twelve cases were excluded as follows: 10 patients with a personal history of ovarian malignancy, 13 cases with recently diagnosed ovarian cancer referred for completion surgery, 14 cases for prophylactic risk-reducing surgery for BRCA or family history (normal ovarian morphology on imaging), one case with final histology that was not conclusive (necrotic ovarian torsion), seven patients declined surgery and did not attend any further follow-up (one suspected malignant and six diagnosed as benign), two died before histology was obtained due to advanced disease, and 65 were diagnosed as benign on imaging but had less than one year of follow up. A total of 200 cases were included as illustrated in Figure 1.

Diagnostic performance of MDM

The MDM imaging discussion correctly identified 123 masses as ovarian cancer (true positive) and 39 ovarian masses as benign (true negative), whilst 36 benign masses were falsely assumed malignant (false positive), and two ovarian cancers were incorrectly diagnosed as benign (false negative). Details of true/false positive and negative cases are illustrated in Table 1.

**Table 1:** True/false-positive and negative MDM imaging ovarian masses diagnostics details *: One was a serous borderline tumour and had a unilateral salpingo-oophorectomy at the unit. Later, she had fertility-preserving staging in the centre. The second case had an ovarian borderline mucinous tumour with an intraepithelial carcinoma staged as IA. MDM: multidisciplinary meeting; US: ultrasound; MRI: magnetic resonance imaging

Number of cases	Details of index diagnostic tests
True positive=123	110 had major surgery by the gynaecological oncology centre with final histology of cancer; 11 cases had tissue diagnosis with a biopsy but no surgery; 2 cases treated surgically by the referring unit as per patient request.
True negative=39	26 cases with benign histology had surgery at referring units after being discharged from the cancer centre MDM; 2 cases preoperatively classified as benign but underwent major surgery by the gynaecological oncology team due to surgical complexity; 9 cases had > 1-year follow-up with no change or with regression of the lesion; 2 cases had < 1-year follow-up due to complete resolution of presenting mass.
False positive=36	35 cases preoperatively classified as malignant had major surgery by the cancer centre with final benign histology. One case was defined as malignant but the patient declined surgery and follow-up imaging (US, MRI) at a 1-year interval demonstrated no change and the diagnosis was consequently downgraded to benign by MDM.
False negative=2^*^	2 presumed benign by MDM and referred to surgery in the referring unit. Final histology was malignant.
Total	200

The MDM imaging review was able to characterise ovarian masses as benign or malignant with a sensitivity of 98.4% (95% CI: 94.3% to 99.8%) and a specificity of 52% (95% CI: 40.2% to 63.7%). The diagnostic performance of our MDM in characterising ovarian masses is shown in Tables 2-3.

**Table 2:** 2x2 contingency table for all ovarian masses operated by the gynaecological oncology team

	Disease Present	Disease Absent	Total Number
Test Positive	123	36	159
Test Negative	2	39	41
Total Number	125	75	200

**Table 3:** MDM diagnostics performance in characterising ovarian masses MDM: multidisciplinary meeting

Diagnostic Performance	Value	95% CI
Sensitivity	98.40%	94.34% to 99.81%
Specificity	52.00%	40.15% to 63.69%
Positive likelihood ratio	2.05	1.62 to 2.60
Negative likelihood ratio	0.03	0.01 to 0.12
Disease prevalence	62.50%	55.39% to 69.23%
Positive predictive value	77.36%	70.06% to 83.61%
Negative predictive value	95.12%	83.47% to 99.40%

We performed a sub-analysis evaluating the diagnostic accuracy in patients with early-stage disease (Stages I and II) where the greatest diagnostic challenges lie. In our entire cohort, 52/200 (26%) malignancies presented in either Stage I or II (48 operated in the centre and four operated in the units), of which 41 cases were Stage I and the remaining nine were Stage II. We note a marginally inferior diagnostic sensitivity of 96% and a specificity of 52% with a PPV of 58.4% (Tables 4-5). Of note, 34 of the false-positive cases were radiologically staged as an early-stage disease, with only one case presenting with radiological suspicion of nodal disease.

**Table 4:** 2x2 contingency table for benign masses assumed as early-stage ovarian malignancy

	Disease Present	Disease Absent	Total
Test Positive	50	36	86
Test Negative	2	39	41
Total	52	75	127

**Table 5:** Sub-analysis of diagnostic accuracy of early-stage disease

Diagnostic Performance	Value	95% CI
Sensitivity	96.15%	86.79% to 99.53%
Specificity	52.00%	40.15% to 63.69%
Positive Likelihood Ratio	2.00	1.57 to 2.55
Negative Likelihood Ratio	0.07	0.02 to 0.29
Positive Predictive Value	58.14%	52.17% to 63.88%
Negative Predictive Value	95.12%	83.12% to 98.72%

False-positive cases

The focus of the study was on the 35 false-positive cases treated in the gynaecological oncology centre. The median age of the women in this group was 54 years (95% CI: 52 to 60), with a range of 31 to 82 years. The most common histological types observed were the serous cystadenoma/adenofibroma and the mucinous cystadenoma. Table 6 illustrates the histological types of the ovarian masses misdiagnosed as malignant and underwent major surgical treatment in the cancer centre. Besides the primary histological diagnosis (Table 6), there were associated histological findings in seven out of 35 (20%) of the cases (1 adenomyosis, 1 Brenner’s tumour, 1 endometrioma, 1 hemorrhagic cyst and para-ovarian cyst, 1 salpingitis, and 2 uterine leiomyomas).

**Table 6:** Histological diagnosis of the false-positive cases who underwent major surgery in the cancer centre

Histopathology	N	%
Ovarian mucinous cystadenoma	11	31%
Ovarian serous cystadenoma/cystadenofibroma	13	37%
Others (1 mature teratoma, 1 benign ancient Schwannoma, 1 endometriosis, 2 ovarian fibromas, 1 steroid cell tumour, 1 microcystic stromal tumour, 1 ovary with abnormal vascular proliferation, 1 salpingitis isthmica nodosa, 2 struma ovarii)	11	31%
Total	35	100.0%

Thirty-four of the women from this cohort who underwent major surgery had been investigated by pelvic MRI. One patient did not have an MRI as contraindicated but she had a pelvic ultrasound and pelvic and abdominal CT scan. All women had a pelvic US prior to the MRI. Twenty-eight out of 35 patients had an abdominal CT scan as well. Seventeen (49%) false-positive cases were diagnosed as borderline tumours and 18 (51%) were presumed invasive malignancies. Table 7 demonstrates the presumed diagnosis for the different ovarian pathologies based on the imaging MDM review. The mean diameter of the ovarian masses was 12 cm (95%: CI 9-15). On MRI, 57% of the masses were multiseptated, 54% had solid parts and 94% had either multiseptation or solid parts. Fifty-three per cent (53%) of the masses had either restricted diffusion or enhancing soft tissue on MRI. The main MRI characteristics are illustrated in Table 8.

**Table 7:** Presumed diagnosis based on imaging MDM review in the false-positive group BOT: borderline ovarian tumours; MDM: multidisciplinary meeting

Final histology	Presumed BOT	Presumed Malignant	Total
Mature cystic teratoma	1	0	1
Mucinous cystadenoma	9	2	11
Ovarian benign ancient Schwannoma	0	1	1
Ovarian endometriosis	0	1	1
Ovarian fibroma	0	2	2
Ovarian serous cystadenofibroma	4	4	8
Ovarian serous cystadenoma	2	3	5
Ovarian steroid cell tumour	0	1	1
Ovarian benign microcystic stromal tumour	0	1	1
Ovarian with unusual vascular proliferation	0	1	1
Salpingitis isthmica nodosa	1	0	1
Struma ovarii	0	2	2
Total	17 (49%)	18 (51%)	35

**Table 8:** Imaging characteristics of the false-positive adnexal masses *: available for 34 cases who had MRI; **: available for 30 cases MRI: magnetic resonance imaging

Main Imaging Characteristics of False-Positive Ovarian Masses Operated by the Cancer Centre Team	Value
Mass largest diameter in cm (range)	1 to 28 cm
Mass largest diameter in cm (Mean: 95%CI)	12 cm (95%: CI 9-15)
Enhancing soft tissue on MRI^*^(percentage)	16/34 (47%)
Restricted diffusion on MRI^*^(percentage)	9/34 (26.5%)
Multiseptation^*^(percentage)	20/35 (57%)
Enlarged lymph nodes^*^(percentage)	1/35 (3%)
Presence of solid part in the mass^*^(percentage)	19/35 (54%)
Bilateral lesions ^*^(percentage)	8/35 (23%)
Cancer antigen (CA) 125 ** median (95% CI)(units/ml)	21 (95%CI: 15-57)

To assess the ultrasound performance in the false-positive group, we applied the ADNEX model by a retrospective collection of sonographic parameters from stored images and reports (images reviewed by author AS). Parameters were available for 24 out of 35 cases. IOTA ADNEX was able to characterise 17% (4/24), 37.5% (9/24), and 54.2% (13/24) of the false-positive cases as benign masses when 10%, 20% and 30% malignancy cut-offs were used respectively. Table 9 illustrates the ADNEX highest relative risks of the different ADNEX classes (benign, borderline ovarian tumour (BOT), stage I cancer, stage II-IV cancer, and metastatic disease) among the 24 false-positive cases with ADNEX parameters available.

**Table 9:** Highest ADNEX relative risk for tumour category ADNEX: Assessment of Different NEoplasias in the AdneXa; BOT: borderline ovarian tumours

	ADNEX Tumor Class					
Highest ADNEX Relative Risk value	Benign	BOT	Stage I invasive	Metastatic	Stage II-IV	Total N (%)
1.4	4	1	0	0	0	5(20.8%)
1.5	0	0	1	0	0	1(4.2%)
1.6	0	2	0	0	0	2(8.3%)
1.7	0	1	0	0	0	1(4.2%)
1.8	0	1	0	0	0	1(4.2%)
1.9	0	0	0	1	0	1(4.2%)
2	0	1	0	0	0	1(4.2%)
2.3	0	2	0	0	0	2(8.3%)
2.4	0	2	0	0	0	2(8.3%)
2.8	0	1	0	0	0	1(4.2%)
3.3	0	0	0	1	0	1(4.2%)
3.7	0	1	0	1	0	2(8.3%)
3.8	0	0	0	1	0	1(4.2%)
4.2	0	1	0	0	0	1(4.2%)
4.3	0	0	0	1	0	1(4.2%)
5.6	0	1	0	0	0	1(4.2%)
	4 (16.7%)	14 (58.3%)	1 (4.2%)	5 (20.8%)	0	24 (100%)

Regarding the two false-negative cases, ADNEX correctly identified one case as a BOT whilst the other was presumed benign with the highest response rate (RR) of 2.3 and 1.3, respectively.

Surgical characteristics and complications in false-positive cases

In our cohort, 29/35 (83%) of major surgeries in the false-positive group were performed by the open technique. Twenty-seven patients (77%) had lymph node sampling. The average operating time was 164 minutes (total of 4389 minutes) and median in-hospital stay was four days.

Only two intraoperative complications occurred and included 1 small bowel injury and one large bowel injury with primary repair for both and no bowel stoma was required. Post-operatively, five out of 35 patients (14%) developed cluster of differentiation 2 (CD2) post-operative morbidity (2 cases with ileus and 3 cases with sepsis required intravenous (IV) antibiotics). There were no CD3 or CD4 post-operative complications. There was no post-operative (90 days) mortality.

## Discussion

This is the first study examining gynaecological oncology MDM diagnostic performance and the outcomes of false-positive cases. To our knowledge, the impact of over-treatment in gynaecological oncology has not been discussed in detail in the literature. This presents a challenge for both the referring hospital and the cancer centre.

This study determined the diagnostic performance of the cancer centre multidisciplinary team (MDT) in characterising ovarian masses. Information derived from various imaging modalities (ultrasound, MRI, CT), tumour markers, and clinical presentation was considered when formulating a probable diagnosis. NHS England has set precise national cancer waiting-time standards, which encompass specific referral and treatment pathways [12]. These pathways for identifying malignancies enable both the correct and timely treatment of the individuals involved and allow cancer centres to meet national targets. The impact of treating benign patients in a gynaecological oncology centre can be significant for patients, leading to over-treatment, as well as exposure to potential complications, including early menopause. The impact on the cancer centre is also significant in terms of operating lists, surgical times, and inpatient stay. These translate into direct and indirect costs for the involved individuals, families, and society. The use of centre resources for non-malignant cases increases the already intense pressure on centres struggling to meet national requirements. This is more highlighted in the current COVID-19 crisis where robust triaging of cancer patients becomes paramount to prevent adverse clinical outcomes. 

Following this study, we adopted various methods to reduce the rate of false-positive diagnoses and prevent unnecessary over-treatment. These include an intraoperative frozen section for cases with a possible borderline or benign diagnosis. A recent large meta-analysis reports the average sensitivity was 94.0% (95% CI 92.0% to 95.5%; range 73% to 100%), and the average specificity was 95.8% (95% CI 92.4% to 97.8%: typical range 81% to 100%) regarding the use of frozen sections [13]. It must be noted that in the case of borderline ovarian tumours, sensitivity and specificity were significantly lower, with one study reporting 76.2% and 88.7%, respectively; the positive predictive value was 66.7% and the negative predictive value was 92.7% [14]. Furthermore, mucinous histology (p < 0.0001, OR=2.03 (1.47-2.81)) and unilateral tumours (p=0.001, OR=2.39 (1.41-4.06)) have been specifically shown to be associated with the misdiagnosis of BOT [15]. In our study, unilateral mucinous tumours (benign mucinous cystadenomas) comprise approximately one-third of all benign ovarian masses misdiagnosed as malignant. In women in whom fertility-sparing is a priority, a two-stage procedure can be offered if imaging and/or frozen section is inconclusive. During the period of our study, frozen sections were not established practice in the Trust.

The greatest diagnostic challenges are represented by early-stage disease limited to the adnexa. MRI has been adopted in many cancer centres as the gold standard for the characterisation of indeterminate ovarian masses. Published literature reports that sensitivity for classifying malignancy is 91%-100% and specificity is 91%-92% [16]. However, distinguishing the subgroup of borderline lesions is significantly more problematic. In a recent meta-analysis studying borderline or malignant ovarian cancer vs. benign ovarian lesions, the pooled likelihood ratio for the occurrence of a positive MRI result was 6.6 (95% CI: 4.7-9.2) and the post-test probability for borderline or malignant diagnosis was 77% (95% CI: 70-82) [17]. The specificity in our study is low (52%), as all benign diagnosed masses with less than one-year follow-up were excluded. We can reasonably expect these cases to be truly benign and, therefore, the specificity would be much higher, at 74%. We need to take on board that cases referred to MDM are cases suspected to be cancer. Therefore, MDM diagnostic performance will be affected by selection bias, and it will not reflect the real diagnostic performance of imaging as clearly benign-looking tumours were already excluded from referrals. This study identified a false negative rate of <5% and a negative predictive value (NPV) of 95%. Interestingly, both cases erroneously diagnosed as benign had borderline histology. This is consistent with the well-known diagnostic challenge of borderline adnexal masses discussed above. Whilst a cancer centre should aspire to maintain an NPV of 100%, so as not to preclude any patient of potentially life-saving treatment, this must be balanced with the risk of high numbers of over-treatment. In selected cases, close interval imaging or a two-stage procedure may be a solution.

In 2014, Van Calster et al. reported a sensitivity and specificity for ADNEX ultrasound model in characterising ovarian masses into benign or malignant of 96.5% and 71.3%, respectively [2]. External validation studies report similar values [18-19]. Retrospective application of ADNEX diagnosed 14 cases as BOT and four, at least (with 10% cut-off), as benign. It is our belief that the implementation of the ADNEX as an integral part of our MDT will decrease the rate of false-positive diagnoses. More importantly, it will assist in identifying the subgroup of borderline tumours that, whilst malignant, will benefit from less-invasive and individualised surgical treatments. Indeed, this study has prompted a change in our clinical practice, including the use of ADNEX for the screening of adnexal lesions, the implementation of frozen sections, and the adjustment of surgical protocol for suspected/diagnosed borderline lesions. As the ADNEX model was only applied retrospectively and was assessed on a small number of patients, this limits the generalisation of our findings, and more research is still required.

## Conclusions

Although an MDM uses a wide array of tests to characterise ovarian masses, false-positive cases of ovarian cancer impose a real challenge to clinical practice. The risk of overtreatment should be balanced against the benefit of a highly sensitive diagnosis. The most common false positives were serous and mucinous cystadenomas. Other tests can be used by MDM to help to reduce the number of false-positive cases of ovarian cancers. This may implement the use of frozen sections for masses restricted to the ovary (stage 1) when imaging diagnosis is less likely. The IOTA ADNEX model may play a role. However, more research is required to confirm this finding. 
